# Supplementary material for: Investigating the dynamics of tax evasion and revenue leakage in somali customs
Source: PLoS One. 2024 Jun 6;19(6):e0303622. doi: 10.1371/journal.pone.0303622 (PMC11156314; doi:10.1371/journal.pone.0303622)
Supplement: Attachment — Submitted filename: Respsonse on comments of Reviewer 3.pdf [file pone.0303622.s002.pdf]

FEEDBACK ON REVIEWERS' REMARKS REGARDING THE ARTICLE

TITLE: Investigating the Dynamics of Tax Evasion and Revenue Leakage in Somali Customs

No.	Concern of the reviewer	Response of the author	Evidence of action taken
1	I like quantitive approach and the PA/RS as a combined concept, PA indicates that A's have the power to act against the P (enabling corruption practices) and RS model discusses the incentive structure to choose corruption. Separating them into different regression equations seems not appropriate, but combining them into a single model that contains the "systemic opportunity structure" and "personal incentive structure" seems a nice explanation for perceived tax evasion. This could also be nicely lined up with other studies on tax compliance that apply the slippery slope model, where the coercive power could facilitate the opportunity structure and the persuasive power the personal incentive structure.	Thank you for your insightful feedback and for highlighting the potential benefits of integrating the Principal-Agent (PA) and Rational Choice (RS) models into a unified analytical framework for our study on tax evasion and revenue leakage in Somali customs.<br><br>Your suggestion to combine these models to simultaneously consider the "systemic opportunity structure" and "personal incentive structure" offers a compelling approach to understanding the complex dynamics of tax evasion and corruption.<br><br>We have taken your advice to heart and have revised our methodology accordingly. Instead of treating the PA and RS models as separate entities within different regression equations, we have reconfigured our analysis to incorporate these concepts into a single, cohesive model. This integrated model now serves as the foundation of our analysis, allowing us to more accurately capture the interplay between systemic factors that provide opportunities for corruption and the individual incentives that drive such behavior.<br><br>We believe that this methodological refinement significantly strengthens our analysis, offering a more comprehensive understanding of the factors contributing to tax evasion and revenue leakage. The combined model allows us to explore the intricate relationship between the opportunities presented by systemic vulnerabilities and the motivations of individuals acting within these systems, providing insights that were not as clearly discernible when examining these elements in isolation.<br><br>We are grateful for your constructive suggestion, which has been instrumental in enhancing the depth and clarity of our study. We look forward to the opportunity for our revised manuscript to be reconsidered for publication, confident that these changes have addressed your concerns and enriched our contribution to the literature on tax compliance and corruption.	See Section 3 of the manuscript: Research framework and hypothesis development
2	Unfortunately, this also leads to my biggest criticism: Your dependent variable does not measure tax evasion, but the individually perceived prevalence of tax evasion. In addition, the measure is drawn from the same survey that is used to identify your independent variables.	We deeply appreciate your thoughtful critique and the opportunity to clarify the methodology and findings of our study, particularly concerning the measurement of tax evasion perceptions and the robustness of our model. Your insights are invaluable in strengthening the rigor and credibility of our research. Allow us to address the concerns you raised:<br><br>We have taken significant steps to refine our study constructs by employing a confirmatory factor analysis (CFA) to ensure the constructs' validity and reliability. This rigorous	See sections 4 and 5

	Therefore, what your model actually measures is the extent in which the perception of the surveyed individuals on tax evasion is coherent with their perception of the system of policies. This would probably explain the high R-squared. (To ensure the high R2 is not caused by overfitting, perform some robustness checks, e.g. by randomly eliminating 15-20% of the observations and repeat this process, then compare the outcomes).	process allowed us to precisely define and differentiate between the perception of tax evasion prevalence and the various systemic factors identified as independent variables. The factor analysis served to reassess and validate the dimensionality of our constructs, ensuring a clear conceptual distinction and reducing the potential for construct overlap. This foundational work underpins our measurement model, strengthening the interpretability and validity of our findings.<br><br>In response to your observation, we have employed Structural Equation Modeling (SEM) to delineate clearly between the measurement model (defining the constructs) and the structural model (explaining the relationships between constructs). This approach allows us to rigorously test the coherence between individuals' perceptions of tax evasion and their views on the policy system, accounting for the complex interplay of perceived prevalence and systemic factors. The use of SEM provides a robust framework for assessing the relationships hypothesized in our study while controlling for potential measurement errors and the endogeneity of the constructs.<br><br>We are confident that these methodological enhancements and the results of our robustness checks address your concerns effectively. Our study's findings offer reliable, valid, and rational insights into the dynamics of tax evasion and revenue leakage.<br><br>We believe that our research contributes meaningfully to the ongoing discourse on tax compliance and policy efficacy, providing a nuanced understanding of the perceptual dynamics at play.<br><br>We are grateful for your constructive feedback, which has been instrumental in refining our study and sharpening our analysis.<br><br>We look forward to your further thoughts on our revised methodology and findings.	
3	Technically the model seems to be not appropriate. The survey questions are manually grouped into "latent variables" but they are not independent, the same question must influence the answer to other questions. For example, CFTEC: weak regulatory enforcement should be directly associated with PIS: strengthening enforcement and penalties and ELRF: enforcement of penalties and sanctions. The two	We greatly appreciate your insightful feedback and the technical concerns you have raised regarding the initial modeling approach in our study on tax evasion and revenue leakage in Somali customs.<br><br>Your detailed observation about the interdependence of survey questions and the potential for latent variables to influence one another highlights a crucial aspect of our analysis that requires careful consideration to accurately capture the complex dynamics of tax compliance behavior.<br><br>In response to your critique, we wish to clarify and confirm that we have indeed transitioned to employing Structural Equation Modeling (SEM) in our revised analysis. This methodological shift was made precisely to address the concerns you've outlined—	See sections 4 and 5

	linear regression models do not take such interactions into account. A structural equation model (SEM) would therefore be more appropriate (ignoring the non-independent measure of tax evasion aforementioned).	namely, the interdependencies among latent variables and the need for a more nuanced analytical approach that can account for these complex relationships.<br><br>SEM allows us to effectively model the interactions between various factors identified in our study, including the "systemic opportunity structure" and "personal incentive structure," as well as their impact on perceived tax evasion and customs revenue leakage. By utilizing SEM, we are not only able to analyze the direct effects of these constructs but also to explore the indirect effects and pathways through which regulatory enforcement, penalties, and sanctions influence tax compliance behavior.<br><br>We are confident that this methodological refinement, as now detailed in our revised manuscript, addresses your concerns and substantially strengthens the quality and relevance of our research.<br><br>We are grateful for your guidance, which has been instrumental in enhancing the rigor and depth of our analysis.<br><br>Thank you once again for your valuable feedback.	
4	Not all is lost, the survey is interesting and performing an exploratory (or possibly confirmatory) factor analysis to assess the "emergence" of the latent variables and explain them in the PA/RS setting would be useful and possibly generalizable. This could provide a framework in which the author can explain how counter TE policies could be designed to become more effective. Please be advised to add information on the role of the respondent as to place the responses properly (e.g. does the person work for the customs, is it a businessperson, or is the respondent a "victim" of TE practices). This could also contribute to a better explanation of the subjective view of the system (policy makers might think they do the right thing because they do not perceive the thing right).	Thank you for your encouraging words and the insightful suggestions regarding the further development of our study on tax evasion (TE) perceptions and their implications for policy design in Somalia. We are grateful for your recognition of the potential value of our survey data and your constructive guidance on enhancing the analytical depth of our research.<br><br>In light of your recommendations, we are pleased to inform you that we have conducted a Confirmatory Factor Analysis (CFA) to assess the emergence and validity of latent variables within the context of the Principal-Agent (PA) and Rent-Seeking (RS) frameworks. This methodological approach has allowed us to rigorously test the structure of our theoretical model and ensure that the constructs developed are indeed reflective of the underlying dimensions of TE perceptions and behaviors.<br><br>The CFA process was instrumental in identifying and validating the key constructs representing systemic opportunity structures, personal incentive structures, and governance deficiencies influencing TE. By confirming the factor structure of our model, we have established a solid foundation for exploring how these constructs interact within the PA/RS setting, thereby offering valuable insights into the mechanisms driving TE and the potential for designing more effective counter-TE policies.<br><br>We believe that these enhancements, informed by your valuable feedback, significantly improve the relevance and potential impact of our research. The refined analysis, now incorporating CFA and a detailed consideration of respondent roles, offers a comprehensive framework for understanding the determinants of TE perceptions and	See sections 4 and 5

	Changing the paper's scope to explain the perception of TE and find what determines such views can be very relevant for future policy design (so perform CFA) and could be publishable. Since you have the data and the framework, I did not reject the paper fully.	designing policies that are both effective and attuned to the diverse needs and perspectives of stakeholders.<br><br>We are confident that the revised manuscript, with its focus on explaining TE perceptions and their policy implications, represents a significant contribution to the field and meets the publication standards of the journal.<br><br>We are grateful for the opportunity to refine our work based on your insightful suggestions and look forward to the possibility of our study contributing to more effective and equitable TE policy design.	
5	A few minor comments: your text has a lot of repetition, which must be reduced. For example, ever sentence in section 2.2.2. claims the same argument: RS is the individual enrichment without creating societal value. In addition, even if the author is spot-on with the policy recommendations, there is no clear scientific argument presented in the paper that backs these recommendations. Therefore, the author should better relate the policy recommendation with the model outcomes and literature review.	Thank you for your valuable feedback on our manuscript. Your insights have been instrumental in identifying areas for improvement, and we have taken careful steps to address these issues in the revised manuscript.<br><br>We take to heart your feedback regarding the connection between our policy recommendations and the outcomes of our model analysis, as well as their grounding in the existing literature. In response, we have significantly enhanced the discussion section of our manuscript to explicitly draw these connections.<br><br>These revisions aim to ensure that our policy recommendations are not only clearly derived from our study's findings but are also comprehensively supported by scientific argumentation and existing literature.<br><br>We believe that these changes significantly enhance the coherence, relevance, and impact of our manuscript.<br><br>We are grateful for your constructive critique, which has been pivotal in strengthening the quality and clarity of our work.<br><br>We look forward to your feedback on the revised manuscript and hope that the modifications we have made address your concerns effectively.	See sections 6
6	Finally, provide the data into a public repository to meet the journal's standards. In addition, since you are using survey data, you need to have an informed consent form the respondents (which I cannot find here) and you need to have an ethics commission reviewing your research. The piece could also gain form some language editing and changes in the structure.	We are grateful for your comprehensive review and final comments, which guide us in refining our manuscript to meet the journal's standards fully.<br><br>Your attention to the crucial aspects of data sharing, ethical considerations, and the overall quality of our manuscript is invaluable. Please allow us to address each point as follows:<br><br>**Public Repository Data Access**:<br>We understand the importance of transparency and replicability in research, especially in studies involving complex analyses and significant implications, such as ours. To this	

		end, we are committed to making our dataset publicly available in alignment with the journal's policies and the broader scientific community's best practices.<br><br>**Ethics Approval and Informed Consent**:<br><br>Ethical considerations and the well-being of our respondents have been paramount throughout our research process. We have obtained an Ethics Approval Certificate from Ethics Committee, Center for Research and Development of SIMAD University, which reviewed and approved our study's design, including the survey methodology and data collection procedures. This certificate confirms our adherence to the highest ethical standards and the protection of participants' rights and confidentiality.<br><br>**Professional Proofreading and Structural Improvements**:<br>To enhance the clarity, coherence, and overall quality of our manuscript, we have engaged the services of a professional proofreading and editing service specializing in academic publications. This comprehensive review has addressed language, grammar, and structural aspects of the manuscript, ensuring that our arguments are articulated clearly and effectively. We have also made targeted revisions to the manuscript structure to improve its flow and readability, ensuring that our findings and recommendations are presented in a logical and accessible manner.<br><br>We believe these actions address your concerns and significantly enhance the manuscript's integrity, accessibility, and scholarly contribution. We are confident that these steps demonstrate our commitment to ethical research practices, data transparency, and the highest standards of academic writing.<br><br>We appreciate your guidance in making these critical improvements and hope that our revised manuscript now fully meets the journal's publication criteria.<br><br>We look forward to your feedback and the opportunity to contribute our research to the journal.<br><br>Thank you once again for your thorough review and constructive suggestions.	
