# Supplementary material for: Investigating the dynamics of tax evasion and revenue leakage in somali customs
Source: PLoS One. 2024 Jun 6;19(6):e0303622. doi: 10.1371/journal.pone.0303622 (PMC11156314; doi:10.1371/journal.pone.0303622)
Supplement: Attachment — Submitted filename: Respsonse on comments of the AcademicEditor.pdf [file pone.0303622.s003.pdf]

FEEDBACK ON REVIEWERS' REMARKS REGARDING THE ARTICLE

TITLE: Investigating the Dynamics of Tax Evasion and Revenue Leakage in Somali Customs

**Concern of the reviewer**	**Response of the author**
Please review your reference list to ensure that it is complete and correct. If you have cited papers that have been retracted, please include the rationale for doing so in the manuscript text, or remove these references and replace them with relevant current references. Any changes to the reference list should be mentioned in the rebuttal letter that accompanies your revised manuscript. If you need to cite a retracted article, indicate the article's retracted status in the References list and also include a citation and full reference for the retraction notice.	Thank you for your careful review and important comments regarding the reference list in our manuscript. We have reviewed our references to ensure that they are both current and accurate, in accordance with the journal's standards.<br><br>Following your recommendation, we have removed all citations and corresponding references to retracted papers from our manuscript. We have also replaced these references with current and relevant literature to maintain the integrity and credibility of our work. The following references have been removed:<br><br>1. Sharma, R., Insurgency And Terrorism. 2023: Guarav book center.<br>2. Arewa, M. and S. Davenport, The Tax and Technology Challenge. Innovations in Tax Compliance: Building Trust, Navigating Politics, and Tailoring Reform, 2022.<br>3. Dom, R., et al., Innovations in tax compliance: Building trust, navigating politics, and tailoring reform. 2022: World Bank Publications.<br>4. Hoadley, M.C. and N. Hatti, Understanding Corruption: Traditional and Legal Rational Norms. 2020: Routledge.<br>5. Coolidge, J. and S. Rose-Ackerman, in African Regimes. 1997.<br>6. Podsakoff, P.M., et al., Common method biases in behavioral research: a critical review of the literature and recommended remedies. Journal of applied psychology, 2003. 88(5): p. 879.<br>7. Cohen, J., Statistical power analysis for the behavioral sciences 2nd ed.(Hillsdale, NJ: L. Erlbaum Associates). 1988.<br><br>Each of these references was scrutinized, and none are now included in our manuscript, ensuring there is no use of retracted or invalid studies. We have updated the manuscript and the reference list to reflect these changes, reinforcing the validity of our research and adhering to ethical publishing standards.<br><br>We hope that these revisions meet the journal's requirements and appreciate the opportunity to enhance the quality of our submission. Thank you once again for your guidance and attention to detail.
